# Pharmacodynamics Must Inform Statistics: An Example from a Cocaine Dependence Pharmacotherapy Trial

**DOI:** 10.1155/2014/927290

**Published:** 2014-02-05

**Authors:** Theresa M. Winhusen, Daniel F. Lewis, Eugene C. Somoza, Paul Horn

**Affiliations:** ^1^Addiction Sciences Division, Department of Psychiatry and Behavioral Neuroscience, University of Cincinnati, College of Medicine, 3131 Harvey Avenue, Cincinnati, OH 45229, USA; ^2^Veterans Affairs Medical Center (VISN 10), 3200 Vine Street, Cincinnati, OH 45220, USA; ^3^Department of Mathematical Sciences, University of Cincinnati, 2693 Campus Drive, Cincinnati, OH 45221, USA; ^4^Division of Neurology, Cincinnati Children's Hospital Medical Center, 3333 Burnet Avenue Cincinnati, OH 45229, USA

## Abstract

*Background.* There is no FDA-approved medication for cocaine dependence or consensus on the statistical approach for analyzing data from cocaine dependence pharmacotherapy trials. The goal of this paper is to illustrate the importance of understanding medication's pharmacodynamics when specifying the statistical model to test its efficacy. *Method.* Data from a double-blind placebo controlled trial of reserpine for cocaine dependence are analyzed. Since the antihypertensive properties of reserpine are well established, blood pressure data are utilized to evaluate the ability of two statistical models, one that does not take the pharmacodynamics of reserpine into account and one that does, to detect reserpine's antihypertensive effect. *Results.* The statistical model specified without regard to reserpine's pharmacodynamics failed to find a significant medication effect for either systolic (*P* = 0.49) or diastolic (*P* = 0.59) blood pressure. Contrariwise, the model based on the pharmacodynamics of reserpine found a significant effect for both systolic (*P* = 0.002) and diastolic (*P* = 0.004) blood pressure. *Conclusions.* If the pharmacodynamics of a study medication are not considered when specifying statistical models, then erroneous conclusions may be reached. This trial is registered with NCT00033033.

## 1. Introduction

According to the Office of National Drug Control Policy, there are over 3 million long-term cocaine users in the USA [1]. Because psychosocial interventions for cocaine dependence are associated with high relapse rates [2, 3], substantial resources have been devoted to finding a pharmacological treatment. Despite these efforts, there is no FDA-approved pharmacological treatment for cocaine dependence. In view of this fact, it seems wise to carefully review the current designs of cocaine pharmacotherapy trials with the goal of identifying and correcting existing inefficiencies to maximize our ability to detect a medication effect if such were to exist in a future clinical trial. The gold standard for determining the efficacy of a medication is the double-blind randomized placebo controlled clinical trial with statistical analysis of the study data. While there is wide-spread agreement that “double-blind” and “randomized” are gold standards for study design there is less agreement on the statistical approach that should be utilized; other than that the statistical plan should be defined a priori.

The trend over the past 15 years has been the use of increasingly sophisticated statistical analytic approaches, about which the study investigators, who have posed the hypotheses to be tested, may have only a superficial understanding. With this increasing gap between the statistical knowledge of the investigators and the analytic approaches being used, there is a danger that investigators will choose to “leave the statistics to the statisticians.” The problem with this approach was summarized in an article entitled “Medical statistics-no time for complacency” in which Aalen [4] outlined several key issues for consideration by medical statisticians. One of these issues is of particular import for investigators conducting pharmacologic trials: “biological and medical knowledge should be strengthened among medical statisticians” [4]. It is important that investigators understand that most statisticians are not, nor should they be expected to be, knowledgeable about the pharmacodynamics of the medications being studied nor of the condition that they are attempting to treat. Without this knowledge, there is a danger that the statistical model being applied to a clinical trial is suboptimal given the phenomenon being studied and, thus, could lead to erroneous conclusions.

We encountered the reality of this possibility in analyzing the results of our double-blind placebo controlled trial of reserpine for cocaine dependence [5]. Reserpine was originally used as an antihypertensive medication and its ability to reduce blood pressure has been well established for over forty years [6]. We were, thus, quite surprised that the protocol-defined statistical analysis did not reveal a significant effect for reserpine on blood pressure. Based on the good medication compliance in the trial, which was greater than the compliance observed in our earlier trial of reserpine, a trial which had shown a significant medication effect on blood pressure [7], we concluded that the lack of observed medication effect must lie in the statistical approach taken. The insights that we obtained through the evaluation of the statistical model are of import for anyone conducting pharmacotherapy trials, particularly in evaluating treatments for disorders for which there is no effective medication and, thus, for which subtle effects are of interest from the vantage of identifying potential mechanisms of action for future study. Within any given statistical approach, several decisions must be made about how best to analyze the data. The present paper examines the import of the pharmacodynamics of a study medication for three aspects of the statistical analysis: (1) whether to include the titration data in the analysis, (2) defining the statistical effect of interest, and (3) defining the terms to include in the statistical model.

## 2. Method

### 2.1. Participants

Vital signs data were collected from 119 participants randomized into a double-blind placebo controlled trial of reserpine for cocaine dependence [5]. Three study sites, located in Boston Massachusetts, Cincinnati Ohio, and Dayton Ohio, recruited participants. All participants were given a thorough explanation of the study and signed an informed consent form that was approved by the Institutional Review Boards and the VA Medical Center Research and Development Committees of the participating sites.

Eligible participants were at least 18 years of age and in good physical health as determined by a medical history, physical exam, electrocardiogram, and standard laboratory tests. Participants were required to have at least one positive urine toxicology screen for the cocaine metabolite benzoylecgonine (BE) (i.e., >300 ng/mL) during the two-week screening period, to meet DSM-IV criteria for cocaine dependence as assessed by the structured clinical interview for DSM-IV (SCID) [8] and to be seeking treatment for cocaine dependence. Participants were excluded from the study if they required detoxification from alcohol, met DSM-IV criteria for dependence on any substance other than cocaine, alcohol, nicotine, or marijuana, or were court-ordered to cocaine dependence treatment. Other exclusion criteria included any serious psychological disorder requiring ongoing treatment such as psychosis or bipolar disorder, a Hamilton Depression score greater than 15, and a history of suicide attempts or current suicidal ideation. Individuals were also excluded if they were currently taking reserpine, had a medical condition that could be exacerbated by reserpine, were taking a medication that could adversely interact with reserpine, or had a known or suspected hypersensitivity to reserpine. Women were ineligible for the study if they were pregnant or unwilling to use an adequate method of birth control.

*Vital Signs Measurement. *During the two-week baseline period and the twelve-week active trial, participants were scheduled to attend three research visits per week. Sitting blood pressure was assessed three times per week during baseline and the first two weeks of the active phase and then weekly thereafter.

### 2.2. Procedures

Study candidates signing informed consent entered the screening and baseline phase which entailed the completion of six clinic visits within two consecutive weeks. Stratified randomization, balancing for gender and self-report of cocaine use (<18 or ≥18 days of use in the last 30 days), was used to assign eligible participants to reserpine or placebo within each study site. All participants were scheduled to receive one tablet per day (i.e., one 0.25 mg tablet of reserpine or one placebo tablet) during dose escalation (week 1) and dose taper (week 13). During weeks 2 through 12, all participants were scheduled to receive two tablets per day (i.e., two placebo tablets or two 0.25 mg tablets of reserpine). During the twelve week active trial participants were scheduled to attend three research visits per week. Study participants received $10 in retail scrip or vouchers per research visit; at the end-of-study evaluation (first visit of week twelve), participants received an additional $25 in retail scrip or vouchers because of the larger assessment burden associated with the visit. A master's level clinician provided each study participant with an hour of individual cognitive behavioral therapy on a weekly basis.

### 2.3. Data Analysis

#### 2.3.1. Pharmacodynamics and Statistical Analysis Overview

There are many elements to consider when defining the analytic approach and statistical model for analyzing data from a cocaine pharmacotherapy trial. Understanding of some of these elements, for example, the occurrence of missing data or the distributions associated with common outcome measures, can be gained through familiarity with the data sets collected from multiple cocaine dependence pharmacotherapy trials. An element that is more specific to a given trial is the study medication being tested, the pharmacodynamics of which need to be considered in order to define an optimal statistical analysis. The investigator needs to consider and to clearly communicate to the statistician what is known about the effective dose of the medication and the speed with which the medication will exert its effect on the outcome variable. In terms of “speed” one could consider medications like methylphenidate, valium, and nicotine patch, all of which begin working almost immediately, at one end of the continuum and drugs like antidepressants, such as SSRIs, tricyclic antidepressants, and MAO inhibitors, which take up to six weeks to exert an effect, at the other end of the continuum.

*Inclusion of Titration Data. *Understanding the effective dose of the medication will also help determine whether or not the data collected during the medication titration phase should be included in the analysis. If a medication does not exert an effect until a certain dose is reached, then including the data collected prior to that point could weaken the ability of the statistical test to detect either a Medication main effect or a Medication-by-Time interaction effect. Conversely, if a medication exerts an effect at relatively low doses and with relatively quick speed, excluding the titration phase data could result in weakening a Medication main effect and very possibly eliminating the chance for a significant Medication-by-Time interaction effect. In cocaine dependence trials, we often cannot specify, with certainty, the effective dose of the medication being studied. Given this uncertainty, if the particular medication being studied has a relatively rapid speed of action, then it would likely be important to include all postrandomization study data, along with data collected during titration.

*Defining the Statistical Effect of Interest and Model Terms.* The speed with which a medication exerts its effect on a given outcome should be considered when defining the statistical effect of interest. Currently, a common method for testing the efficacy of a medication in a clinical trial is to compare the impact of medication on the participants' respective rates of improvement (or deterioration) during treatment. In a regression, this translates into determining efficacy solely on the basis of the Medication-by-Time interaction effect. However, it is important to note that even when a medication has a substantial effect, that effect is unlikely to continue increasing indefinitely over time. At some point, the effect would very probably reach a maximum and level off. If this plateau occurs too early in the participants' treatment, then tests based solely on a linear Medication-by-Time interaction would fail to detect the medication effect, whereas an evaluation of the Medication main effect would reveal the efficacy of the medication. When investigators are unable to reasonably rule out the possibility of a medication reaching its peak effect early in the treatment phase, the Medication main effect and the Medication-by-Time interaction effect can be evaluated jointly to determine efficacy. A significant Medication-by-Time effect would indicate medication efficacy, and in the reasonably certain absence of a Medication-by-Time effect, a significant Medication effect would also indicate efficacy. Of course, basing efficacy on two hypotheses instead of one can reduce statistical power in that one should adjust the alpha level to correct for multiple comparisons; Bonferroni would suggest alpha levels of 0.025 for each hypothesis rather than usual alpha of 0.05 [9].

If the Medication main effect is of interest, then one needs to consider how to include baseline data in the model. For outcome measures with significant intrasubject variability, including a summary of baseline values as a covariate can significantly reduce the standard error for a Medication main effect, thus increasing the statistical power of the test. It is important to note that this reduction in standard error occurs even in cases where the two treatment groups did not differ during baseline. Including baseline as a covariate also helps to mitigate possible imperfections in randomization.

#### 2.3.2. Pharmacodynamics of Reserpine

Reserpine destabilizes the membranes of the vesicles which store catecholamines in neurons. As a result, the storage vesicles rupture causing the neurotransmitters (e.g., dopamine and norepinephrine) to spill out into the cytoplasm and to be metabolized by the enzyme monoamine oxidase (MAO). This is an immediate and irreversible effect of reserpine even at the 0.25 mg dose [10]. No new dopamine is available to be discharged into the synaptic junction until new vesicles become available. This requires protein synthesis, which takes several days to weeks. During this period, individuals exposed to reserpine hypothetically would not experience the euphoric effects of cocaine, as it would not be possible for cocaine to augment the synaptic concentration of dopamine in the nucleus accumbens. This forms the rationale for theorizing that reserpine might lead to a decrease in cocaine use, since it would not be reinforcing during this period (and for as long as patients continue to take reserpine). Reserpine reduces blood pressure through the same mechanism.

#### 2.3.3. Statistical Model to Evaluate Reserpine's Effect

The protocol-specified statistical analysis plan for the reserpine trial called for the use of generalized estimating equations (GEE) [11, 12] to compare the reserpine and placebo groups on the data following medication titration (weeks 2–12). This GEE model included medication, week, and the Medication-by-Week interaction effect, with the Medication-by-Week interaction being the effect of interest. It is important to note that this statistical analysis is reasonable when the pharmacodynamics of reserpine are not considered. The statistical model based on a consideration of the pharmacodynamics of reserpine differs in several important ways. In this alternative model, the GEE model included the study data from weeks 1 through 12 and included baseline as a covariate and the following terms: Medication, Week, and the Medication-by-Week interaction effect, with the Medication main effect and Medication-by-Week interaction effect viewed jointly as the statistical effects of interest. Since reserpine's antihypertensive action is well established, we were able to test the validity of the two statistical approaches based on their ability to detect reserpine's effect on systolic blood pressure (SBP) and diastolic blood pressure (DBP).

## 3. Results

### 3.1. Participant Characteristics

There were no significant baseline demographic [5] or blood pressure differences between the study groups. The study sample primarily consisted of African American male crack users who were generally high school graduates. Approximately half worked full or part time and approximately half were or had been married. The mean age of the sample was 41.0 (SD = 7.6). At baseline, the mean SBP and DBP were 124.5 (SD = 11.3) and 79.4 (SD = 9.0), respectively, which places the sample, on average, in the prehypertension range [13].

### 3.2. Inclusion of Titration Data in the Statistical Model

The protocol-specified statistical analysis Plan called for excluding the data collected during the one-week titration period. As stated above, deciding on the inclusion of the titration phase data needs to be based on information regarding the effective medication dose as well as the relative speed with which the medication manifests an effect on the outcome of interest. While we were not entirely certain of the effective dose of reserpine, as pertains to its hypothesized effects on cocaine use, we were certain that its effect would be fairly rapid (see Section 2.3.2 above). From this perspective, it is clear that the titration data should be included in the analysis. The effect of this single decision point on the analysis of the SBP and DBP can be seen in Table 1, which provides the results from the statistical analysis when the one-week titration phase is or is not included. As can be seen, inclusion of the one-week titration period changes the Medication main effect *P* value from 0.35 to 0.11 in the case of SBP and from 0.23 to 0.08 in the case of DBP. Somewhat interestingly, the *P* values for the Medication-by-Week interaction effects actually increase by including the titration data. This increase could reflect the fact that, all else being equal, if there is, in fact, no Medication-by-Week interaction effect (i.e., the medication effect was rapid), adding data points is more likely to yield a result that reflects the true lack of a Medication-by-Week interaction effect. This, of course, reinforces the importance of correctly specifying the effect of interest.

### 3.3. Statistical Effect(s) of Interest and Terms Included in the Statistical Model

**Figure 1:**
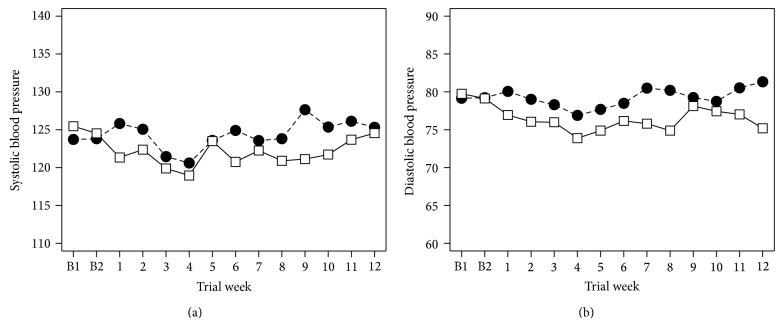
Mean (a) systolic and (b) diastolic blood pressure as a function of medication group and study week. –∙– Placebo; —□— reserpine.

For the reserpine trial, the analytic plan specified that the Medication-by-Week interaction effect, which provides information about the change in the treatment groups over time, was the effect of interest. The graphs of the SBP and DBP data can be found in Figure 1. As can be seen, reserpine decreased blood pressure during week 1 and maintained that decrease during the remainder of the trial. As discussed above, a Medication-by-Time interaction effect is very likely to miss a medication's efficacy in this type of situation.

In addition, the analytic plan specified that baseline data not be included as a covariate unless the groups differed significantly at baseline. However, blood pressure has relatively high intrasubject variability and, thus, inclusion of baseline as a covariate can significantly reduce standard error even in cases where the groups do not differ significantly from one another during baseline. The impact of including baseline as a covariate and adding the Medication main effect as an effect of interest can be seen in Table 2. If one were to review the results of the analysis that did not include baseline as a covariate, and that specified the Medication-by-Time interaction effect as the sole effect of interest, one would most certainly conclude that reserpine had no significant effect on either DBP or SBP. In reviewing the results of the alternate model, which was specified in accordance with the pharmacodynamics of reserpine, one would conclude, rightly, that reserpine significantly decreased SBP (*P* = 0.002) and DBP (*P* = 0.004).

### 3.4. Original Statistical Model Compared to Pharmacodynamic-Based Statistical Model

**Figure 2:**
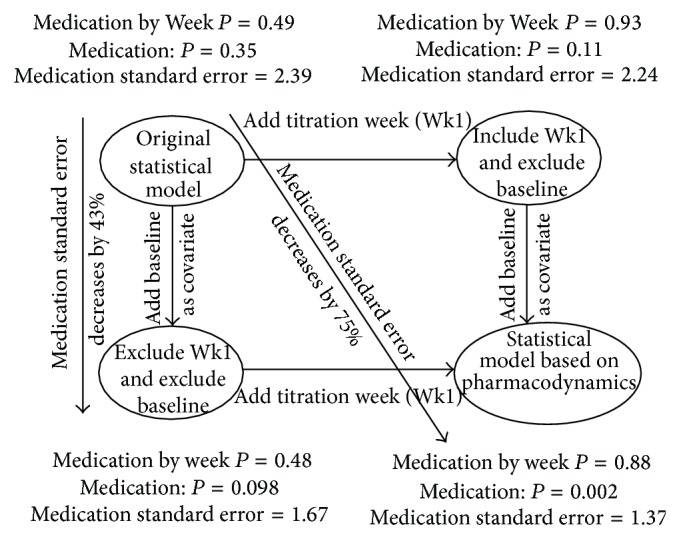
Systolic blood pressure *P* values for the Medication-by-Week interaction and Medication main effect and standard error for Medication main effect as a function of including titration week and baseline as a covariate in the statistical model.

The complete set of differences between the original statistical model and the version derived from a consideration of the pharmacodynamics of reserpine is illustrated in the results for SBP in Figure 2. The results for DBP (data not shown) are very similar to those for SBP. Figure 2 displays the *P* values for the Medication-by-Week interaction and Medication main effects as well as the standard error for the Medication main effect as a function of including the titration week and baseline as a covariate. The impact of some of the differences between the original statistical model, such as adding titration week and baseline as a covariate, is discussed above as well as in Tables 1 and 2. In addition, Figure 2 shows that simply adding baseline as a covariate to the original statistical model, without including the titration week, results in a 43% decrease in the standard error for the Medication main effect and a change in the Medication main effect *P* value from 0.35 to 0.098. Figure 2 also highlights the fact that the standard error for the Medication main effect is decreased by 75% in the pharmacodynamics-based statistical model compared to the original statistical model.

## 4. Discussion

This paper highlights the importance of considering a medication's pharmacodynamics when specifying the statistical model used to analyze pharmacotherapy trial data, with a specific focus on deciding whether to include titration data in the analysis, specifying the effect of interest, and specifying terms to include in the model. As demonstrated by the analysis of reserpine's effect on SBP and DBP, a statistical model that is specified without knowledge of the study medication's pharmacodynamics can lead to a type II error. This finding is consistent with Aalen's [4] observation that a closer connection between medical knowledge and statistical models is needed.

The primary potential limitation of the present analysis involves statistically, relative to clinically, significant effects. Specifically, this paper demonstrates that a statistically significant medication effect can be missed if the statistical model is not based on medication's pharmacodynamics but does not address the degree to which a clinically significant effect might be missed. In the present case, comparing the baseline-corrected final blood pressure readings for the reserpine and placebo groups revealed a difference of 3.0 mm Hg for SBP and 6.9 mm Hg for DBP. While these differences appear small, studies suggest that a 5-6 mm Hg decrease in DBP is associated with a clinically significant reduction in stroke and coronary heart disease even among “normotensive” individuals [14]. This suggests that the statistical model defined without regard to reserpine's pharmacodynamics missed an effect of potential clinical import. In any case, given the costs associated with conducting a clinical trial, it would seem prudent to develop a statistical model that will be as sensitive as possible to potential medication effects and one simple step towards achieving this goal would be to define the model in accordance with the pharmacodynamics of the study medication.

While the focus of the present paper is somewhat circumspect, its implications are not: we cannot afford to conduct research in which there is a disconnect between the study investigators posing the general hypotheses to be tested and the statisticians who are defining, through the statistical models, very specific hypotheses to be tested that may or may not correspond to the hypotheses that should be tested based on knowledge of the medication's pharmacodynamics. It is therefore up to the investigator to provide this knowledge to the statistician and for the statistician to explain the statistical analysis to the investigator in order to ensure that he or she understands precisely what the analysis is testing and for this understanding to be reflected in the a priori analysis plan. Failure of the investigator and statistician to cooperate effectively could result in type II errors in cases, unlike reserpine's effect on blood pressure, where this error cannot be detected.

## Figures and Tables

**Table 1:** Systolic and diastolic blood pressure results as a function of titration data inclusion in the statistical model.

Parameter	Analysis excluding the one week titration phase					Analysis including the one week titration phase				
	Estimate	SE	95% confid. limits	*Z*	*P*	Estimate	SE	95% confid. limits	*Z*	*P*
Systolic blood pressure										
Intercept	123.68	1.94	119.87–127.48	63.66	<0.0001	124.80	1.74	121.38–128.21	71.66	<0.0001
Medication	−2.22	2.39	−6.91–2.47	−0.93	0.35	−3.54	2.24	−7.94–0.86	−1.58	0.11
Week	0.17	0.14	−0.11–0.45	1.22	0.22	0.01	0.13	−0.26–0.27	0.01	0.99
Med. by Week	−0.14	0.21	−0.54–0.26	−0.69	0.49	0.02	0.19	−0.36–0.40	0.09	0.93

Diastolic blood pressure										
Intercept	78.48	1.51	75.52–81.45	51.82	<0.0001	79.33	1.28	76.82–81.84	61.84	<0.0001
Medication	−2.38	1.99	−6.27–1.52	−1.20	0.23	−2.91	1.66	−6.16–0.34	−1.75	0.08
Week	0.09	0.13	−0.17–0.35	0.67	0.51	−0.04	0.11	−0.25–0.17	−0.38	0.70
Med. by Week	−0.11	0.20	−0.50–0.28	−0.54	0.59	−0.03	0.16	−0.35–0.28	−0.18	0.85

**Table 2:** Systolic and diastolic blood pressure results as a function of inclusion of baseline as a covariate in the statistical model.

Parameter	Analysis not including baseline as a covariate^a^					Analysis with baseline as a covariate^a^				
	Estimate	SE	95% confid. limits	*Z*	*P*	Estimate	SE	95% confid. limits	*Z*	*P*
Systolic blood pressure										
Intercept	124.80	1.74	121.38–128.21	71.66	<0.0001	28.10	6.67	15.0–41.19	4.20	<0.0001
Baseline	NA	NA	NA	NA	NA	0.78	0.05	0.67–0.88	14.51	<0.0001
Medication	−3.54	2.24	−7.94–0.86	−1.58	0.11	−4.16	1.37	−6.84–1.48	−3.04	0.002
Week	0.01	0.13	−0.26–0.27	0.01	0.99	−0.0193	0.13	−0.28–0.24	−0.15	0.88
Med by week	0.02	0.19	−0.36–0.40	0.09	0.93	0.02	0.19	−0.37–0.39	0.07	0.94

Diastolic blood pressure										
Intercept	79.33	1.28	76.82–81.84	61.84	<0.0001	20.59	4.26	12.25–28.94	4.84	<0.0001
Baseline	NA	NA	NA	NA	NA	0.74	0.05	0.64–0.84	13.99	<0.0001
Medication	−2.91	1.66	−6.16–0.34	−1.75	0.08	−3.03	1.06	−5.11– −0.96	−2.87	0.004
Week	−0.04	0.11	−0.25–0.17	−0.38	0.70	−0.04	0.11	−0.25–0.16	−0.42	0.67
Med by week	−0.03	0.16	−0.35–0.28	−0.18	0.85	−0.05	0.16	−0.36–0.26	−0.30	0.76

^a^Data from study weeks 1–12 included in analysis.
